# A Flexible and Optical Transparent Metasurface Absorber with Broadband RCS Reduction Characteristics

**DOI:** 10.3390/nano14181507

**Published:** 2024-09-17

**Authors:** Babar Hayat, Jinling Zhang, Adil Khan, Syed Muzahir Abbas, Abdul Majeed, Samir Salem Al-Bawri

**Affiliations:** 1 School of Electronic Engineering, Beijing University of Posts and Telecommunication, Beijing 100876, China; 2 School of Information Engineering, Xi’an Eurasia University, Xi’an 710065, China; 3 School of Engineering, Faculty of Science and Engineering, Macquarie University, Sydney, NSW 2109, Australia; 4 Space Science Centre, Climate Change Institute, Universiti Kebangsaan Malaysia (UKM), Bangi 43600, Malaysia

**Keywords:** absorber, transparent, flexible, broadband, RCS reduction

## Abstract

Metasurface absorbers (MSAs) are of significant importance in a wide range of applications, such as in the field of stealth technology. Nevertheless, conventional designs demonstrate limited flexible characteristics and a lack of transparency, hence constraining their suitability for certain radar stealth applications. This study introduces a novel MSA operating in the broad microwave range, which exhibits both optical transparency and flexibility. The structure consists of a flexible substrate made of polyvinyl chloride (PVC), along with a resistive film composed of indium tin oxide (ITO). The proposed structure exhibits the ability to effectively absorb over 90% of the energy carried by incident electromagnetic (EM) waves across the frequency range of 9.85–41.76 GHz within an angular range of 0° to 60°. In addition, to assess the efficacy of the absorption performance, an examination of the radar cross-section (RCS) characteristics is conducted. The results indicate a reduction of over 10 dB across the aforementioned broad frequency spectrum, regardless of the central angle.

## 1. Introduction

In recent years, there has been a surge of interest among scientists and engineers in the field of microwave technology. This area of study has gained significant importance due to its crucial role in various applications, including radar systems, imaging techniques, and wireless communications. However, the system’s quality is jeopardized by pervasive EM interferences and noises. In order to limit the EM jamming, metasurface-based absorbers are utilized [1,2]. Metasurfaces (MS), which are the two-dimensional equivalent of metamaterials, consist of artificially engineered periodic or aperiodic subwavelength structures [3,4]. They attract a lot of attention because of their remarkable EM manipulation properties for numerous applications, such as RCS reduction [5,6], radome design [7,8], and antenna design [9,10]. In particular, the introduction of MS has provided a new perspective on electromagnetic absorption (EMA) and led to the development of unique design methodologies for radar absorbers. In comparison to conventional absorbers, such as the Salisbury screen [11], resistive frequency selective surface [12], Jaumann absorber [13], circuit analog absorber [14], and magnetic absorbers [15], absorbers based on metasurfaces offer several advantages, including a low-profile design, cost-effectiveness, high absorptivity, and a simplified design process. Subsequently, a multitude of MSAs have been developed and examined, covering microwave [16], terahertz [17], infrared, and visible bands [18]. These absorbers can exhibit single-band, dual-band, or multi-band features.

Nevertheless, important limitations of the majority of MSAs are their limited bandwidth and lack of polarization stability, which pose challenges for their practical implementation. Recently, many technologies have been employed to achieve broadband absorption. These include the utilization of a multilayered structure [19], a single-layer absorber structure consisting of multi-resonance units with varying geometric dimensions [20], and the use of lumped components loading technology [21]. Yet, the real-world deployment of MSAs is limited due to the inflexibility and hardness of solid and stiff dielectric materials [22]. Curved platforms with variable packing and shape are commonly employed in practice, mostly in domains where aerodynamics and aesthetics are important, such as high-speed airplanes, missiles, and vehicles [23,24]. Also, these structures provide difficulties due to their complex design, heavy weight, and poor visual visibility. The crucial element in the development of a flexible absorber with optical transparency lies in the utilization of a flexible dielectric substrate, such as polydimethylsiloxane (PDMS), polyvinyl chloride (PVC), or polyimide (PI) sheet, when used with an indium tin oxide (ITO) sheet [25,26,27]. Amongst these flexible dielectric substrates, PDMS and PVC have minimal dielectric losses as compared to PI, resulting in better absorption efficiency. ITO finds extensive application in the field of transparent and flexible electronic devices due to its remarkable electrical and optical characteristics. The lower the surface resistance, the more closely its properties resemble those of metals [28]. To this day, there exists a limited number of studies that have been undertaken about the development of transparent wideband flexible absorbers. For example, a transparent and flexible MSA constructed from a PVC substrate and an ITO film was suggested by Cheng et al. [29]. The MSA is severely constrained in its bandwidth and polarization. In addition, by creating a multilayer structure, Yao et al. [30] were able to enhance the capability of backward scattering suppression in broadband. However, the absorber’s performance in terms of optical transparency is lacking.

In addressing the aforementioned problems, this paper introduces a microwave-frequency broadband MSA that possesses remarkable optical transparency and flexibility. The fundamental element is a closed-bracket-shaped design made of indium tin oxide (ITO) and printed on flexible transparent substrates that include polyvinyl chloride (PVC) and polyethylene terephthalate (PET). A broadband absorption over 90% is achieved within the frequency range of 9.85 to 41.76 GHz, having a relative absorption bandwidth of 124%. The proposed absorber is polarization-insensitive over a wide incidence angle of 60° in either polarization direction. Additionally, an RCS reduction of above 10 dB is observed across the specified frequency range even for curved surfaces. Ultimately, the flexible metasurface absorber (FMSA) was fabricated and a favorable correlation was seen between the numerical and experimental results. This outcome further strengthens the reliability and validity of our design. When compared to prior reports, this particular structure exhibits numerous advantages, including enhanced ultra-broadband capabilities, a low profile, stability over a wide range of incident angles, optical transparency, and curvature-insensitive performance.

## 2. Design and Results

**Figure 1:**
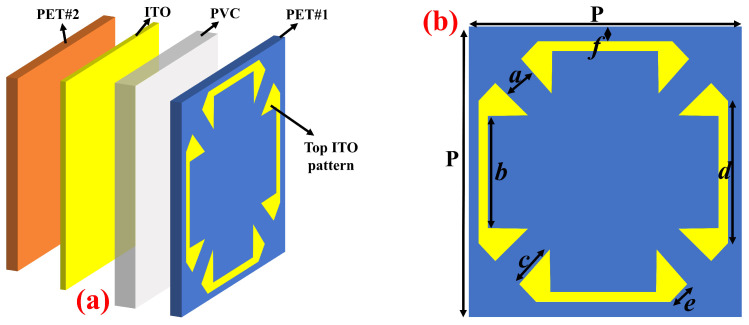
Schematic geometry of a unit cell. (**a**) The 3-D view, and (**b**) top ITO pattern.

Figure 1a depicts the schematic diagram of the proposed FMSA unit cell configuration. The structure comprises two layers of resistive-ITO material, which are then filled with a flexible dielectric substrate. The yellow component of the structure is composed of ITO with a sheet resistance of 170 ohm/square $\left(\Omega /□\right)$ and a thickness of 20 nm. The uppermost ITO pattern exhibits a configuration like a square bracket shown in Figure 1b, whereas a continuous ITO sheet is present at the bottom. The supporting layers for resistive-ITO sheets are composed of PET ($\varepsilon_{r}=3$, tanδ=0.06) films with a thickness of 0.125 mm. The PVC ($\varepsilon_{r}=2.7$, tanδ=0.001) was chosen as a flexible substrate with 1.6 mm thickness, allowing it to be used in conformal applications. The permittivity of PET and PVC used in this study was measured at resonant frequency, i.e., 25 GHz. The proposed structure is simulated and optimized using the frequency domain solver of the commercially available Microwave Suite 2020. The lateral directions employ unit cell boundary conditions, whereas an open boundary condition is specified in the z-direction. The EM waves are incident upon the structure in the negative z-direction by a Floquet port, with its electric field polarized along the *y*-axis. The simulation utilized a tetrahedron optical mesh size of 18,002, with a minimum edge length of 0.01 and a maximum edge length of 12. This mesh size enables a precise depiction of the structure and accurate calculation of the electromagnetic field interactions. The optimized parameters of the unit cell are as follows: P=8, a=1, b=3, c=1.3, d=3.6, e=0.9, f=0.2 (units: mm).

**Figure 2:**
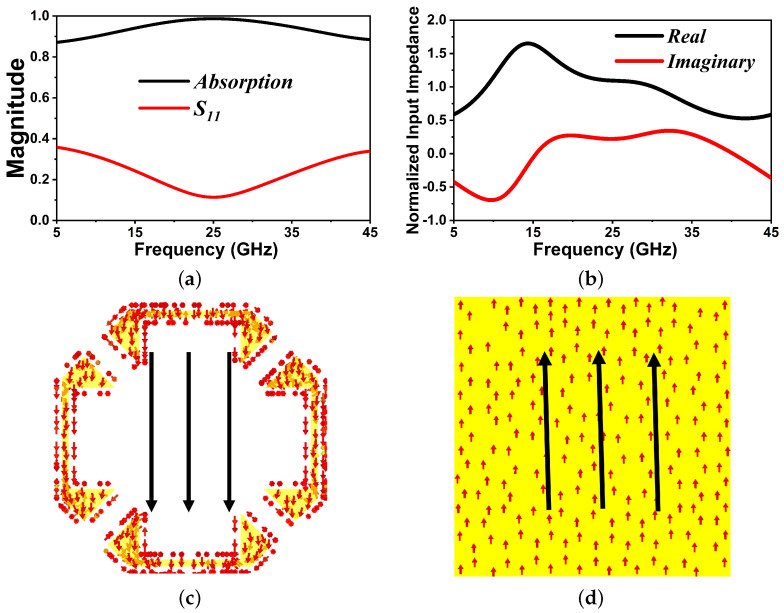
(**a**) Simulated reflection coefficient and absorptivity spectrum of the proposed FMSA. (**b**) Normalized input impedance. Surface current distribution on (**c**) top and (**d**) bottom layer at 26 GHz.

**Figure 3:**
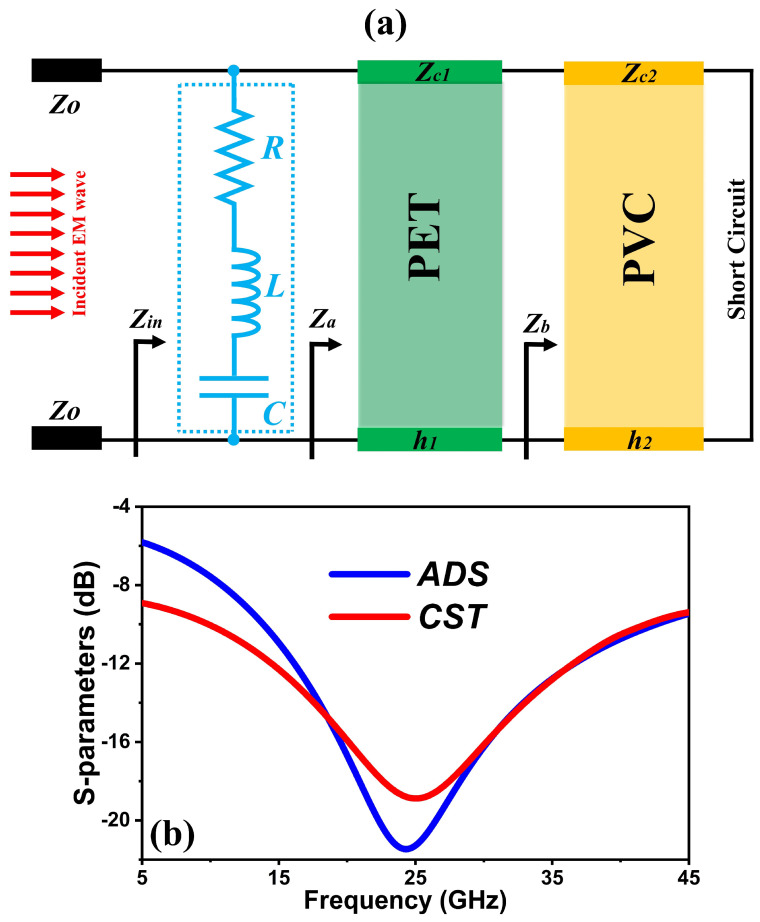
(**a**) Schematic diagram of ECM in ADS; (**b**) The S-parameters from ADS and CST simulations.

The absorptivity A(ω) of a presented FMSA can be determined by analyzing its S-parameters as:

(1) $$A\left(\omega \right)=1-T\left(\omega \right)-R\left(\omega \right)=1-S_{21}^{2}-S_{11}^{2}$$

where $T\left(\omega \right)=S_{21}^{2}$ and $R\left(\omega \right)=S_{11}^{2}$ are the transmissivity and reflectance, respectively. By employing continuous ITO film as the bottom layer, the suggested design achieves nearly zero transmissivity. Thus, the absorptivity A(ω) of the design can be calculated as $A\left(\omega \right)=1-R\left(\omega \right)=1-S_{11}^{2}$. The absorption spectra of the FMSA under normal incidence waves are shown in Figure 2a. It has been shown that the structure exhibits an energy absorption rate of over 90% within the frequency range of 9.85 to 41.76 GHz. In contrast, the element exhibits the ability to function across a broad range of frequencies without the occurrence of multiple resonances. This is due to the inclusion of a lossy pattern, which significantly reduces the overall quality factor of the structure [29]. Figure 2b illustrates the normalized input impedance (*Z*) of a proposed absorber, providing further understanding of its absorptivity performance. The FMSA’s impedance is well matched to that of free space over a large frequency range (i.e., 9.85 to 41.76 GHz), resulting in a perfect interface absorption [29,30,31,32]. Furthermore, an additional factor contributing to the significant absorption seen is magnetic resonance induced by the antiparallel surface current at the top pattern and ground surface, as illustrated in Figure 2c,d. Based on transmission line theory, an equivalent circuit model (ECM) is modeled to assess the ultra-wideband absorption performance of the FMSA as previously reported in [33,34]. The ECM schematic representation is shown in Figure 3a. The free-space impedance is $Z_{0}=377\;\Omega .\;Zc_{1}=Z_{0}/\sqrt{\varepsilon_{1}}$ and $Zc_{2}=Z_{0}/\sqrt{\varepsilon_{2}}$ are the characteristic impedances of the transmission line, which depend on the dielectric constants of PET and PVC, respectively. The circuit is terminated by a short circuit in order to simulate the ground plane because of the continuous ITO film. The top layer with resistive-ITO patterns is represented as a series capacitance *C*, resistance *R*, and inductance *L* circuit connected in parallel. Therefore, its impedance is proved as [1]:

(2) $$Z=R+j\omega L+\frac{1}{j\omega C}$$

According to transmission line theory, the input impedance of a short-circuited transmission line is defined as [33,34]:

(3) $$Z_{a}=Z_{c1}\frac{Z_{b}+jZ_{c1}\tan\left(\beta_{1}h_{1}\right)}{Z_{c1}+jZ_{b}\tan\left(\beta_{1}h_{1}\right)}$$

(4) $$Z_{b}=jZ_{c2}\tan\left(\beta_{2}h_{2}\right)$$

Here, $Z_{c1}$, $\beta_{1}$, and $h_{1}$ are the characteristic impedance, phase constant, and thickness of the PET dielectric substrate, respectively. Whereas, $Z_{c2}$, $\beta_{2}$, and $h_{2}$ are the characteristic impedance, phase constant, and thickness of the PVC with the ITO plane, respectively. Now, the input impedance $\left(Z_{in}\right)$ of the whole structure is shown by the following equation:

(5) $$Z_{in}=Z\;\|\;Z_{b}$$

Finally, the calculation of the reflection coefficient can be determined as follows:

(6) $$\Gamma =\frac{Z_{in}-Z_{o}}{Z_{in}+Z_{o}}$$

In order to verify the reliability of the analysis, we provide the comparison results between the calculation of the equivalent circuit model and the CST simulation, as illustrated in Figure 3b. The developed ECM of the FMSA is simulated using Keysight’s advanced design system (ADS). The values of the components are as follows: R=10 Ω, L=0.245 nH, C=0.175 pF. The reflection coefficient of the equivalent circuit is steady with the simulation outcome from CST Studio Suite 2020.

**Figure 4:**
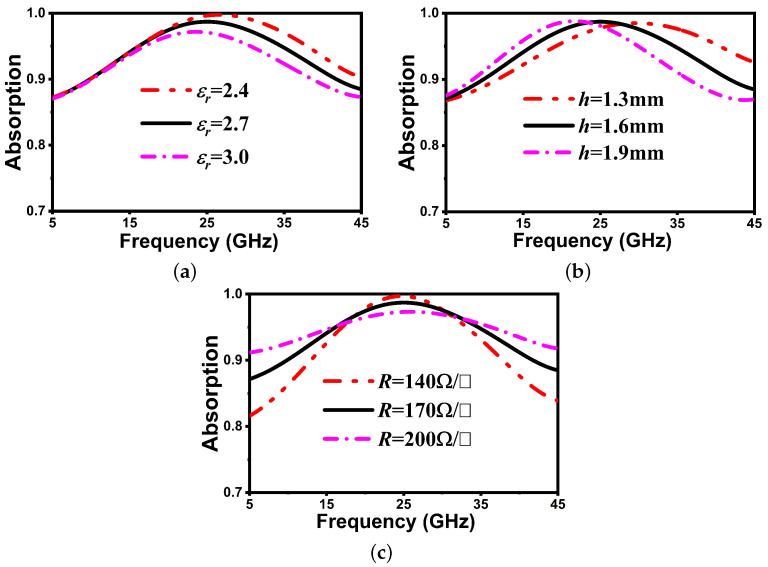
Absorption curves by varying (**a**) dielectric constant, (**b**) PVC thickness, and (**c**) ITO resistance.

Also, it is necessary to evaluate the impact of the element characteristics and shape on attaining maximum absorption within a specific frequency range. Figure 4a investigates the impact of different relative permittivity values of the PVC substrate on the absorptivity. It is evident that the absorption band shows a shift toward the lower spectrum as the $\varepsilon_{r}$ values are increased. Hence, the absorption frequency band can be adjusted by varying the dielectric constant of the flexible PVC substrate. In addition, it can be observed that the absorptivity decreases with increase in the permittivity. This is because, as the permittivity increases, the induced polarization within the material becomes stronger. This stronger polarization tends to reflect more of the incident wave rather than allowing it to penetrate deeper into the material. As a result, less energy is absorbed by the material from the incident wave, leading to a decrease in absorptivity. Another important parameter that influences the absorptivity is the flexible substrate thickness. It is one of the most important criteria in absorber design as it determines the flexibility and low-profile character of the proposed structure. According to Maxwell’s principle of scale invariance, there is an inverse relationship between substrate thickness and resonant frequency. As can be seen in Figure 4b, the back frequency shift is realized with a reduction in bandwidth when a substrate’s thickness is increased. Further, Figure 4c illustrates the impact of changing the sheet resistance (R) on both the efficiency and bandwidth of the absorber. It has been observed that increasing the sheet resistance increases the absorption bandwidth. However, the efficiency suffers significantly as a consequence. This is because of the sheet resistance’s influence on impedance matching. Therefore, to keep a balance between the bandwidth and efficiency, the optimal value of the sheet resistance is chosen as 170 Ω/□. Overall, a substrate permittivity of 2.7 and thickness of 1.6 mm along with a sheet resistance of 170 Ω/□ are selected for designing the FMSA presented in this paper, as they provide ultra-wideband absorbing performance with high efficiency. In practical applications, EM waves meet the surface of a structure at various angles; therefore, the polarization-angle insensitivity is another predicted performance metric.

**Figure 5:**
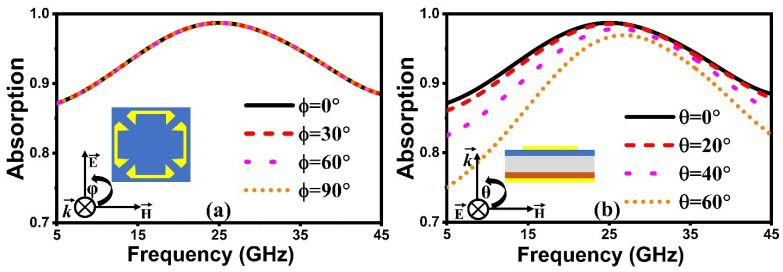
Simulated absorption response at distinct (**a**) polarization angles, and (**b**) incident angles.

Figure 5a illustrates the spectra of absorptivity for different polarization angles ranging from 0° to 90° while keeping a normal incidence angle. Based on the symmetric structure of the proposed FMSA, the absorptivity remains constant from 9.85 to 41.76 GHz, indicating that the absorber has the advantage of being polarization-insensitive. Furthermore, an investigation of the absorptivity performance at different incident angles is also carried out, as depicted in Figure 5b. The findings demonstrate that the absorption efficiency degrades slightly with varying the incident angles. This is due to the interaction between the wave’s electric and magnetic fields with the structure’s surface. Overall, the capabilities of our design across a desired spectrum are highly effective within an angular span of 0° to 60°. This structure is well suited to use in microwave applications due to its polarization-independent and wide incidence angle stable characteristics. It is well known that numerous stealth structures, such as ships, radomes, and jets, are not planar surfaces. To verify the efficacy of a proposed FSMA for stealth applications, a grid consisting of 30 × 30 unit cells is constructed and the RCS properties are examined for both planar and non-planar designs. For comparison, a copper plate with a thickness of 0.035 mm and an electric conductivity of 5.8 × 10^7^ S/m is designed to be similar in shape and size to the proposed absorber. The Studio Suite was used to conduct full-wave simulations using a horizontally polarized plane EM wave that was incident along the negative *z*-axis at a distance of λ/4 from the MS.

**Figure 6:**
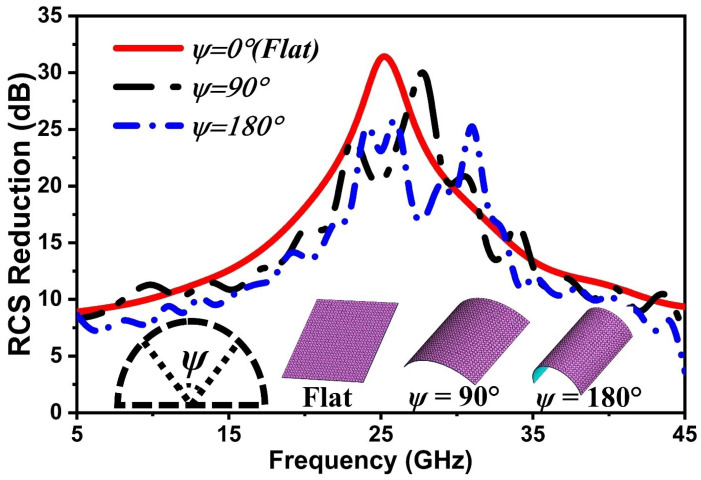
RCS reduction curves for different ψ.

Figure 6 illustrates the simulated RCS reduction for planar and conformal FMSA. In the case of flat $\left(\psi =0\right)$, the RCS reduction of above 10 dB is realized throughout a broad frequency range of absorptivity, ranging from 9.85 to 41.76 GHz. Also, for the defined frequency range, it is clear that curved surfaces $\left(\psi =90{}^{\circ}\;\mathrm{and}\;\psi =180{}^{\circ}\right)$ can maintain an RCS reduction of over 10 dB. However, a minor alteration for lowering the radar cross-section (RCS) is observed on surfaces with curvature. This occurs because the incident angle of EM waves varies with the curvature of a surface. In general, our design shows a significant decrease in RCS across multiple arrangements, rendering it well suited for a wide range of stealth applications.

## 3. Experimental Verification

Finally, to validate our theoretical study, we fabricated an FMSA with dimensions of 240 × 240 × 1.6 mm^3^ for experimental purposes. The upper ITO structure with a sheet resistance of 170 Ω/□ is created by using laser etching on the PET substrate (PET# 1 in Figure 1), while the lower ITO film with a similar sheet resistance is deposited on another PET layer (PET# 2 in Figure 1) using magnetron sputtering, with a processing accuracy of 5 μm.

**Figure 7:**
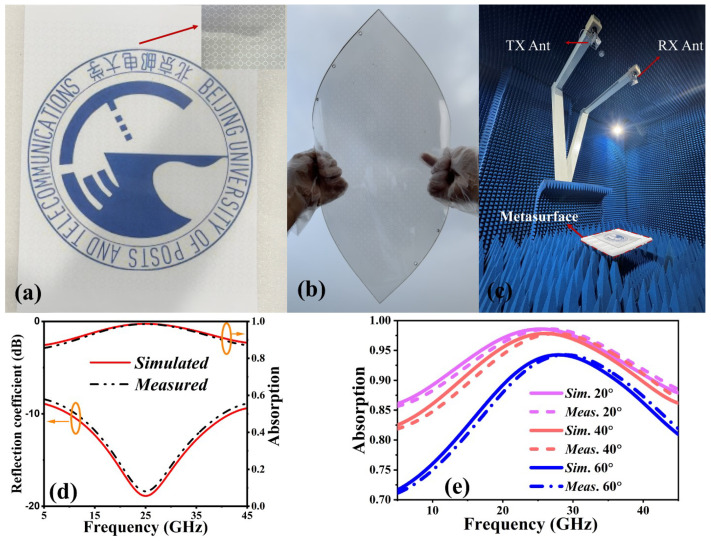
(**a**) Photograph of the sample and the details of a unit cell shown in inset. (**b**) The flexibility of the sample. (**c**) Experimental setup. (**d**) Simulated and measured results of S11 (left) and absorption (right) at normal incidence. (**e**) Simulated and measured absorption under oblique incidence.

Following that, a thin layer of transparent glue is used to interlayer the top and bottom PET with flexible PVC. Figure 7a depicts the prepared sample that was designed, exhibiting details in the inset. Additionally, the optical transmittance of a sample is illustrated by positioning the university monogram behind it, making it readily apparent to the human eye. Figure 7b depicts the sample in the state of bending, providing evidence of its excellent flexibility. In addition, an anechoic chamber is used for measuring the reflection coefficient of the sample. The setup includes two pairs of wideband horn antennas with a frequency range of 5 to 18 GHz and 18 to 45 GHz. These antennas are linked to an Agilent Network Analyzer (VNA) model N5234A, as shown in Figure 7c. The sample is placed in front of the horn antennas surrounded by absorbing materials. Both the sample and horn antennas have their centers adjusted to the same height. Furthermore, the reflectivity of the FMSA is standardized in comparison to a metal plate of similar dimensions. Consequently, the absorption can be obtained by subtracting the square of the magnitude of $S_{11}$ from 1, as expressed by the equation $A=1-{\left|S_{11}\right|}^{2}$. Figure 7d illustrates the measured reflectivity and absorptivity at normal incidence. A wide absorption bandwidth is displayed by the FMSA, which exhibits 90% absorption between 10.21 and 40.3 GHz. The slight difference between the simulated and measured absorption bandwidths is mainly caused by the tolerances in fabrication and assembly; measurement errors and the variation in permittivity of the substrates or resin films that are used to combine different layers are not taken into account in the element simulation. In addition, the absorptivity maintains values above 80% across the whole intended spectral range even when the incident angle is varied up to 60°, as illustrated in Figure 7e. The experimental results demonstrate a strong correlation with the simulated results, providing evidence that the suggested absorber can effectively decrease backward reflection and show its potential use in conformal applications. Table 1 presents a comparison between the suggested absorber and other recently published studies. It clearly demonstrates that our design outperforms earlier structures in terms of flexibility, bandwidth, low profile, single layer, compact size, and insensitivity to a wide range of incident wave angles.

## 4. Conclusions

This paper presents a microwave absorber-based FMSA array for stealth applications. An ITO-PET-PVC unit cell is employed to achieve a wide range of absorption over 90% from 9.85 to 41.76 GHz owing to the magnetic resonance, with measured optical transmittance of 74%. Additionally, after conducting a comprehensive evaluation of the RCS reduction capability, we determined that the FMSA presented in this study is capable of achieving an RCS reduction of over 10 dB in both planar and conformal configurations, with a relative bandwidth of 124%. The simulated results were validated by experiments, confirming that the FMSA demonstrates both wideband absorptivity and angular stability. The utilization of flexible materials, such as PET and PVC, makes the designed structure appropriate for microwave stealth and can easily be modified for various frequency ranges.

## Figures and Tables

**Table 1:** Comparison with reported state-of-the-art work.

Ref	Size (mm²)	No. of Layers	Thickness (λ)	Bandwidth (%)	Flexible	Angular Stability (%)	RCS Reduction
[24]	6 × 6	4	0.11	128	No	45	Planar
[25]	10 × 10	4	0.96	158	No	50	-
[26]	16.6 × 16.6	1	0.48	124	No	40	-
[29]	5 × 5	1	0.08	40	Yes	45	Planar and conformal
[30]	4 × 4	2	0.33	129	Yes	50	-
Mine	8 × 8	1	0.13	124	Yes	60	Planar and conformal

## Data Availability

The data that support the findings of this study are available from the corresponding author upon reasonable request.
